# Gorham–Stout disease: good results of bisphosphonate treatment in 6 of 7 patients

**DOI:** 10.1080/17453674.2019.1709716

**Published:** 2020-01-13

**Authors:** Kristian Nikolaus Schneider, Max Masthoff, Georg Gosheger, Sebastian Klingebiel, Dominik Schorn, Julian Röder, Tim Vogler, Moritz Wildgruber, Dimosthenis Andreou

**Affiliations:** a Department of Orthopaedics and Tumor Orthopaedics, University Hospital of Münster, Germany;; b Institute of Clinical Radiology, University Hospital of Münster, Germany

## Abstract

Background and purpose — Gorham–Stout disease (GSD) is a rare mono- or polyostotic condition characterized by idiopathic intraosseous proliferation of angiomatous structures resulting in progressive destruction and resorption of bone. Little is known about the course of disease and no previous study has evaluated patients’ quality of life (QoL).

Patients and methods — This is a retrospective analysis of 7 consecutive patients (5 males) with a median age at diagnosis of 14 years and a median follow-up of 7 years who were diagnosed with GSD in our department between 1995 and 2018. Data regarding clinical, radiographic, and histopathological features, and treatment, as well as sequelae and their subsequent therapy, were obtained. QoL was assessed by Musculoskeletal Tumor Society Score (MSTS), Toronto Extremity Salvage Score (TESS), and Reintegration to Normal Living (RNL) Index.

Results — 3 patients had a monoostotic and 4 patients a polyostotic disease. Besides a diagnostic biopsy, 4 of the 7 patients had to undergo 8 surgeries to treat evolving sequelae. Using an off-label therapy with bisphosphonates in 6 patients, a stable disease state was achieved in 5 patients after a median of 20 months. The median MSTS, TESS, and RNL Index at last follow-up was between 87% and 79%.

Interpretation — Due to its rare occurrence, diagnosis and treatment of GSD remain challenging. Off-label treatment with bisphosphonates appears to lead to a stable disease state in the majority of patients. QoL varies depending on the individual manifestations but good to excellent results can be achieved even in complex polyostotic cases with a history of possibly life-threatening sequelae.

Gorham–Stout disease (GSD) is a rare mono- or polyostotic condition characterized by the idiopathic intraosseous proliferation of angiomatous structures, resulting in progressive destruction and resorption of bone (Rauh and Gross 1997, Boyer et al. 2005, Dellinger et al. 2014). Gorham and Stout (1955) specified the condition concluding that “the progressive osteolysis is always associated with an angiomatosis of blood and sometimes of lymphatic vessels, which seemingly are responsible for it.”

Today, around 300 cases of GSD have been described. Clinical manifestations depend on the affected site as well as on evolving sequelae, like bone deformity, spontaneous fractures, pericardial effusion, chyloperitoneum, or chylothorax due to leaks in the lymphatic vessels network or thoracic duct invasion (Patrick 1976, Tie et al. 1994, Ludwig et al. 2016). The diagnosis of GSD can be difficult and laboratory findings are usually normal (Liu et al. 2016). Local radiographs may initially demonstrate unspecific patchy radiolucencies (Adams et al. 2016, Ramaroli et al. 2019), while progressive bone osteolysis can be observed later on (Kuriyama et al. 2010; Dellinger et al. 2014). The final diagnosis is based on histopathological examination of a biopsy specimen of the affected bones (Dellinger et al. 2014, Zanelli et al. 2020).

There have been isolated reports in the literature describing cases where an osteolysis stopped progressing after several to many years (Boyer et al. 2005). Treatment options include systemic treatment with bisphosphonates, sirolimus and interferon alpha-2b (IFNα-2b), radiation, and local surgery but no gold standard has yet emerged (Hammer et al. 2005, Heyd et al. 2011, Li et al. 2018, Mo et al. 2018, Ramaroli et al. 2019).

We evaluated the long-term disease course of patients with GSD focusing on clinical disease features, treatment including bisphosphonates, and sequelae, as well as patients’ QoL using a combination of standardized scoring systems (Tunn et al. 2008).

## Patients and methods

7 consecutive patients (5 males) who were diagnosed with GSD in our department between 1995 and 2018 were included in this study. All patients were symptomatic and/or had documented disease progression. Anonymized pertinent data were obtained from patients’ records. Patients’ quality of life at last follow-up was determined using a combination of standardized scoring systems, as proposed by Tunn et al. (2008): Musculoskeletal Tumor Society Score (MSTS), Toronto Extremity Salvage Score (TESS), and Reintegration to Normal Living (RNL) Index.

The median age at diagnosis was 14 years (5–42) and the median follow-up amounted to 7 years (1–22). The median time from first symptoms to final diagnosis was 27 months (3–60). All patients complained of local pain without history of previous trauma, combined with local swelling in 3 cases, restricted range of movement in 1, case and weight loss in another case. 4 patients had a polyostotic and 3 patients a monoostotic disease (Table 1). The diagnosis was histopathologically confirmed in all patients. Biopsies showed dilated thin-walled lymphatic and vascular vessels within rarefied lamellar bone (Figure 1).

Figure 1.Dilated thin-walled lymphatic and vascular vessels within rarefied lamellar bone.

**Figure:**
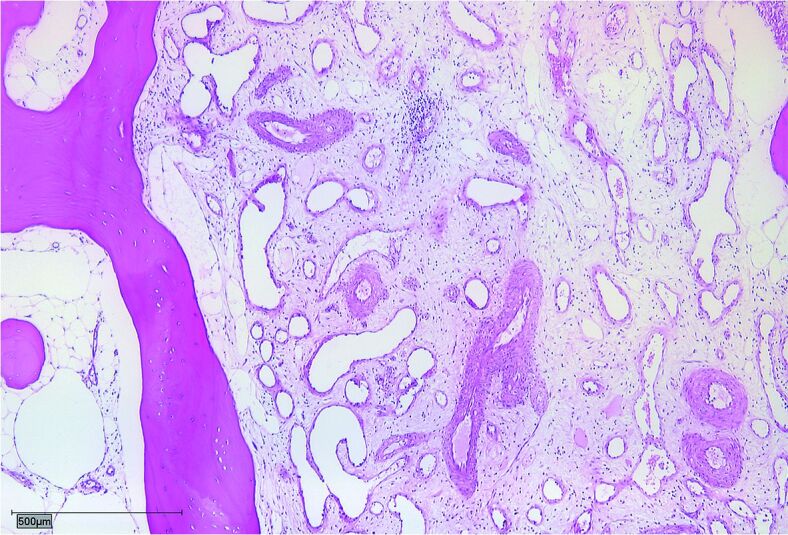

**Figure:**
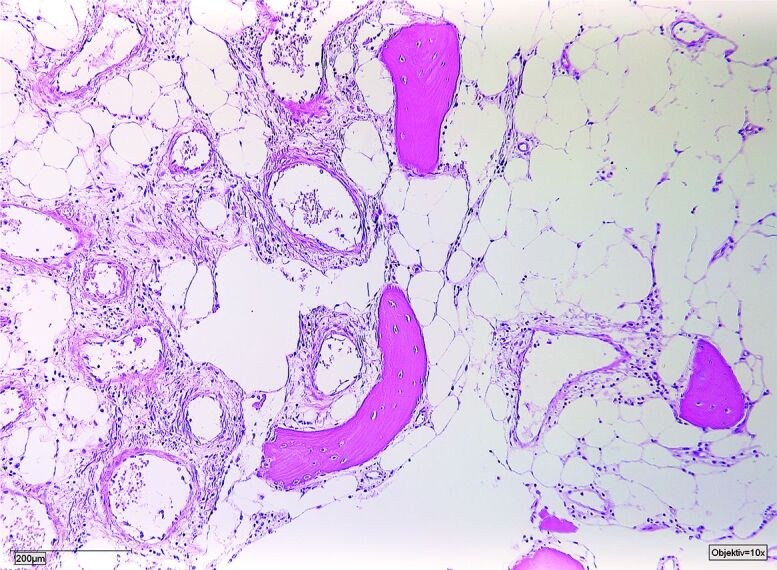

**Table 1.** Clinical data of 7 patients with GSD

Patient	Sex	Age diagnosed	Follow-up	Initial symptoms	Involved bones
1	Male	5y7m	7y3m	Pain, swelling	Skull, thoracic and lumbar vertebrae, ribs, RL pelvis, RL femur, RL humerus
2	Female	29y1m	9y3m	Pain	R ilium
3	Male	21y2m	2y3m	Pain, swelling	RL femur, L pelvis, R humerus, thoracic and lumbar vertebrae
4	Male	13y8m	21y10m	Pain	Thoracic and lumbar vertebrae, sternum, RL pelvis, R humerus
5	Female	11y7m	8m	Pain, swelling	L tibia
6	Male	42y7m	6y10m	Pain, shoulder impingement	L scapula
7	Male	12y4m	4y7m	Pain, weight loss	Cervical vertebrae

## Results

Local surgical treatment was required in 4 patients (Table 2). 1 patient underwent plate fixation after a femoral fracture (Figure 2), while another patient required occipitocervical fusion due to a progressive atlantoaxial instability (Figure 3). A stabilizing spinal instrumentation (T6–L4) was necessary in a third patient due to progressive kyphosis. The same patient required further surgical treatment 4 years later due to progressive osteolysis of T11 and L5 leading to cerebrospinal fluid leaks. Another revision surgery was necessary 5 years later following a rod fracture due to material fatigue. The remaining patient suffered from recurrent erysipelas in his left leg, which was severely deformed from the disease (Figure 4). During an erysipelas bout he developed a life-threatening sepsis, which rendered a knee disarticulation necessary.

Figure 2.Patient no 1, 11-year-old boy with pathologic left distal femoral fracture with wide, dense metaphyseal bands corresponding to continuous 5-year bisphosphonate therapy (a); fracture consolidation 6 weeks postoperatively (b).

**Figure:**
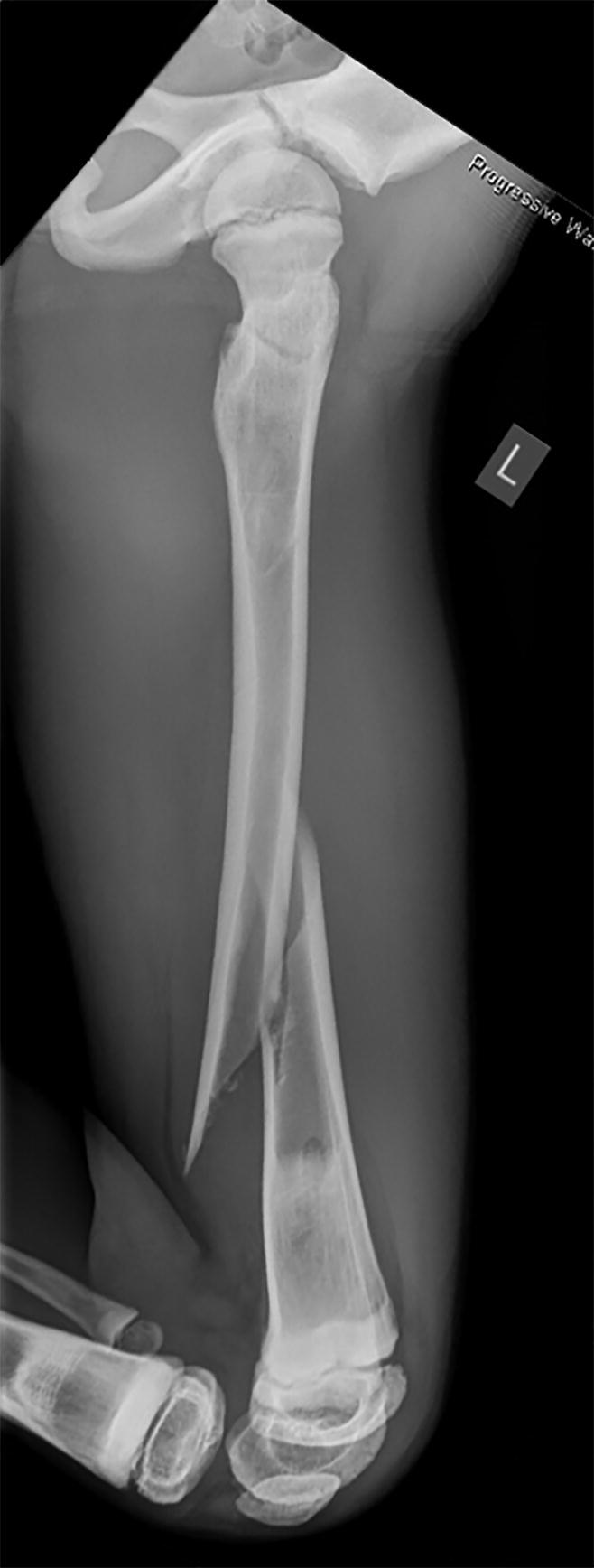

**Figure:**
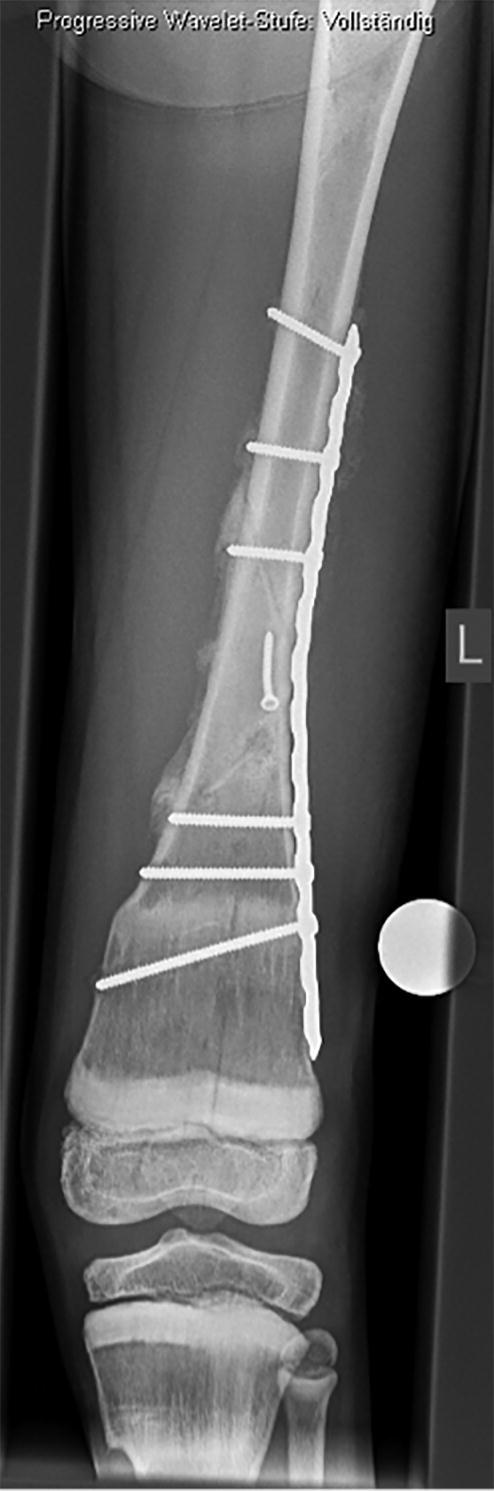

Figure 3.Patient no 7, 13-year-old boy with progressive atlantoaxial instability with multiple GSD lesions in C1–C7 (a); 6 weeks after occipitocervical fusion (b).

**Figure:**
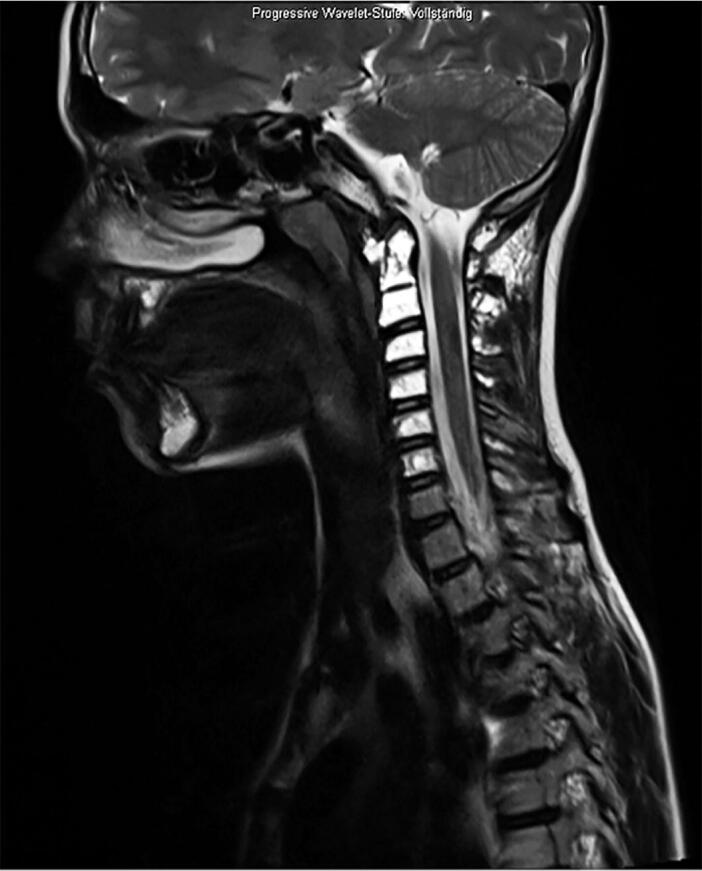

**Figure:**
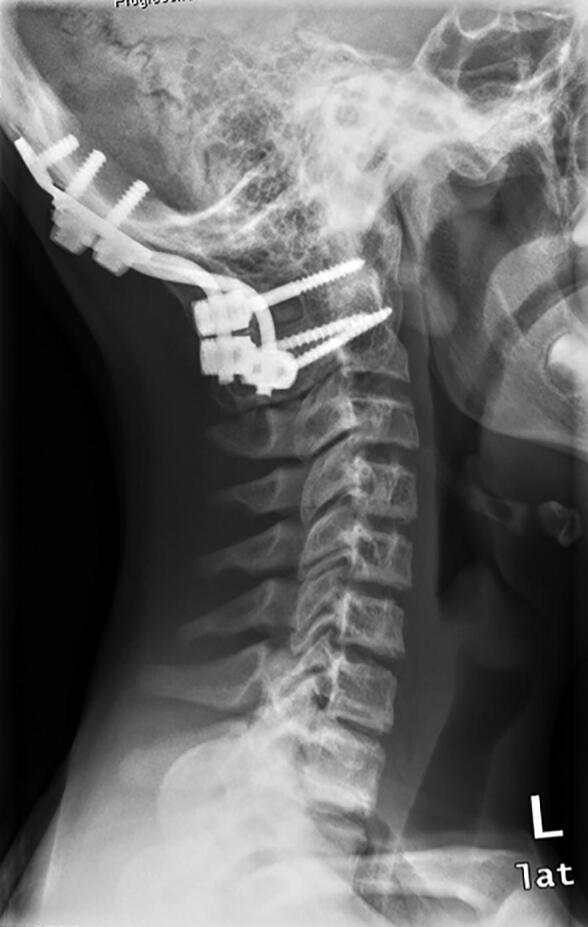

Figure 4.Patient no 5, 12-year-old girl with severely deformed left lower extremity (a) with proliferations of angiomatous structures within bone, muscle, and subcutaneous tissue (b).

**Figure:**
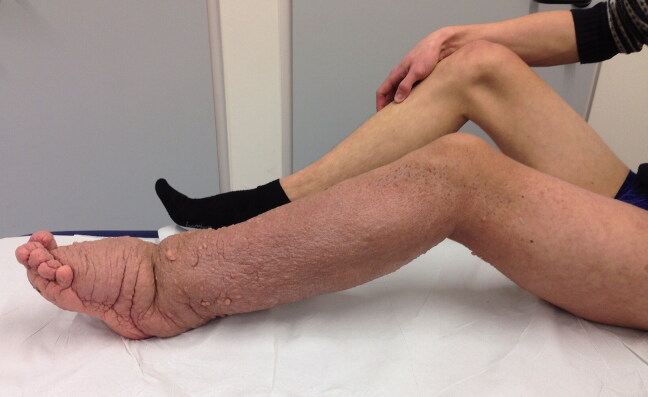

**Figure:**
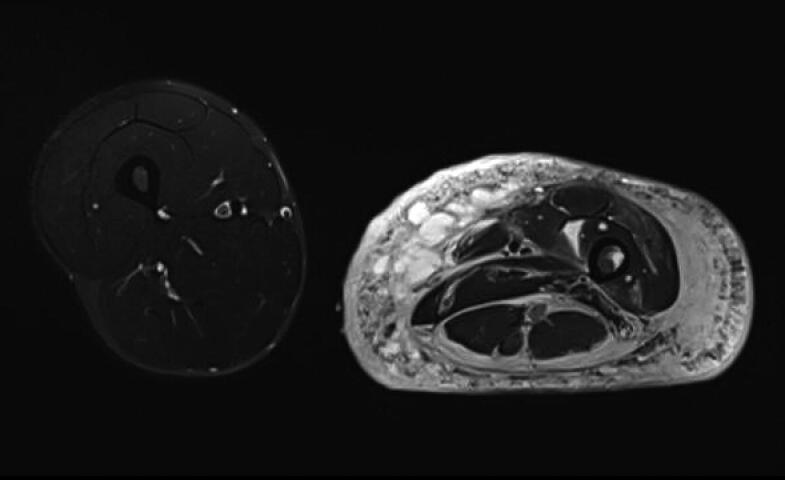

**Table 2.** Clinical data of 7 patients with GSD

Patient	A	B	C	Medication	D	Chylothorax
1	3m	1m	12m	08/11—today: zoledronic acid 2 mg every 4 weeks until 03/18, since 03/18: every 6 weeks 02/10—today: cholecalciferol 1–2 x 1000 IE daily 02/10—today: Ca 2 x 500 mg for 10d after zoledronic acid IV 03/17—07/17 sirolimus 0.5 mg x 2 daily –> discontinued due to recurrent aphthous ulcerations 07/15—09/17 propranolol 1 mg per kg of body weight –> discontinued due to bradycardia	Plate osteosynthesis due to pathologic distal femoral fracture L (5y5m)	R 4y after GSD diagnosis
2	2y	18m	20m	12/10—06/11 zoledronic acid 4 mg every 4 weeks –> discontinued due to incompliance	–	–
3	5y	–	–	Due to a severe nephropathy, bisphosphonate treatment is currently contraindicated in this patient	L knee disarticulation due to recurrent erysipelas and sepsis (0m)	–
4	3y	13y10m	3y6m	09/09—09/10 peginterferon alpha2b 50µg every week –> discontinued due to hyperthyreosis 09/10—06/12 sirolimus 1 mg daily –> discontinued due to progressive chylothorax 01/13—today: sirolimus 1–2 mg daily 02/10—today: zoledronic acid 4 mg every 4 weeks 02/10—today: cholecalciferol 1–2 x 1000 IE daily 02/10—today: Ca 2 x 500mg for 10d after zoledronic acid IV	Spinal instrumentation (T6–L4) (12y7m) Augmented closure of cerebrospinal fluid leaks T11 and L5 (16y3m) Revision surgery after after rod fracture in (22y)	L 14y after GSD diagnosis **^a^**
5	3m	1m	–	02/18—today: zoledronic acid 2 mg every 4 weeks	–	–
6	3y	1m	8m	01/12—today: alendronic acid 70 mg every week	–	–
7	2y	1m	20m	03/14—today: zoledronic acid 4mg every 4 weeks	Occipitocervical fusion (O–C2) due to atlantoaxial instability (8m)	–

A. Time from first symptoms until diagnosis.

B. Time from diagnosis until start of medication.

C. Time of medication until stable disease.

D. Required surgery (time after initial diagnosis)

**^a^**Chylothorax surgery: pleurodesis 10/2011, pleurectomy 03/2015

**Figure 5.**
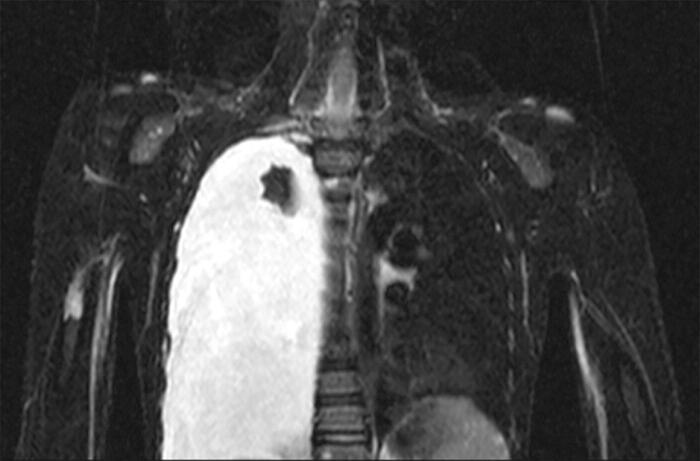
Patient no 1, boy at age 9 year with asymptomatic right chylothorax 4 years after initial diagnosis of GSD.

2 patients developed a chylothorax 4 and 14 years after initial diagnosis, respectively (Figure 5). Surgical treatment with pleurodesis followed by pleurectomy was required in one of these patients due to progressive respiratory distress, while the other patient remains asymptomatic with a stable chylothorax for over 5 years. No patient underwent radiation treatment.

6 patients received bisphosphonate therapy, which was contraindicated in the remaining patient due to a severe nephropathy (Table 2). No adverse effects were observed under bisphosphonate treatment. Stable disease (defined as no radiographic progression of bone resorption) was achieved in 5 patients after a median of 20 months (8–42). 1 patient has been under bisphosphonate treatment for only 2 months with no radiographic follow-up as yet. 2 patients with chylothorax underwent an additional treatment with sirolimus and 1 patient an initial IFNa-2b therapy that had to be discontinued due to hyperthyreosis after 12 months of treatment (Table 2).

The median MSTS at last follow-up amounted to 87% (23–97); the median TESS was 87% (43–97) while the median RNL Index amounted to 79% (39–88). Although the small number of cases in our cohort precluded the use of statistical analyses, we observed that younger patients tended to achieve better score results than patients aged 18 years and older, while male patients fared slightly better than female patients. On the other hand, we found that patients with a monoostotic and polyostotic disease had similar scores. The 2 patients who developed a chylothorax had a somewhat lower MSTS but a similar TESS and RNL Index compared with the patients without chylothorax. Good to excellent results in all 3 QoL scores were achieved in the 6 patients who received bisphosphonates whilst poor results were obtained in the patient who was not able to undergo bisphosphonate treatment (Table 3).

**Table 3.** Median values of QoL scores of 7 patients with GSD

	MSTS	TESS	RNL index
Male sex	87	93	85
Female sex	83	85	77.5
< 18 years old	93	93	85
> 18 years old	77	84.5	77
Monoostotic disease	87	87	79
Polyostotic disease	80	87.5	80
No bisphosphonate treatment	23	43	39
Bisphosphonate treatment	90	90	82

MSTS – Musculoskeletal Tumor Society Score

TESS – Toronto Extremity Salvage Score

RNL – Reintegration to Normal Living

## Discussion

GSD is a rare disease and severe symptoms have been described at presentation, such as bone deformities, pathologic fractures, or neurological deficits due to vertebral osteolysis (Kulenkampff et al. 1990). However, most patients appear to initially develop unspecific symptoms such as pain, regional swelling, or a restricted range of motion (Möller et al. 1999, Patel 2005, Dellinger et al. 2014, Agyeman et al. 2017). We have observed similar initial symptoms in our patients as well (Table 1). As a result, medical consultation, imaging studies and diagnosis may be delayed, with only 2 of our patients being diagnosed within 3 months after developing complaints.

Due to disease progression and local symptoms, 6 of our 7 patients underwent bisphosphonate treatment, and 5 of them achieved radiologically stable disease, while treatment was only recently started in the remaining patient. Several case reports have demonstrated the clinical benefit from bisphosphonates (Mignogna et al. 2005, Yerganyan et al. 2015, Brance 2017, Ramaroli et al. 2019). Hagberg et al. (1997) reported the first successful treatment of GSD with bisphosphonates. Following an unsuccessful radiation treatment, they initiated a bisphosphonate treatment with clodronic acid in combination with IFNa-2b in a 19-year-old male patient who suffered from progressive, polyostotic disease with a chylothorax, and achieved a “rapid improvement” of the patient’s general condition. Hammer et al. (2005) later reported that bisphosphonate monotherapy in GSD resulted in an “immediate clinical improvement” and radiologically stable disease within two years in a 45-year-old woman.

Despite the activity and clinical benefits in GSD, various possible adverse effects of bisphosphonate treatment have been described. Besides gastrointestinal intolerance, flu-like symptoms (after intravenous application) and the development of osteonecrosis of the jaw (ONJ) have been described (Reid 2011, Wessel et al. 2008). Despite the fact that we have administered a frequent bisphosphonate regime to the majority of patients and the average treatment duration amounted to over 4 years, none of our patients developed an ONJ or other relevant adverse effects in long-term follow-up. Informed consent to off-label usages is required as bisphosphonates are not approved by the U.S. Food and Drug Administration (FDA) or the European Medicines Agency (EMA) for treatment of GSD (Dellinger et al. 2014).

Another treatment option for GSD patients reported in the literature is sirolimus, an mTOR inhibitor that acts as a down-regulator of cellular proliferation and angiogenesis (García et al. 2016). García et al. (2016) described a successful sirolimus treatment in a 43-year-old female patient with GSD of her left hemithorax accompanied by a left-sided chylothorax. Despite achieving remission within 4 weeks, treatment had to be discontinued due to metrorrhagia. We initiated sirolimus treatment additionally to bisphosphonate treatment in our 2 patients who developed a chylothorax. However, 1 patient developed recurrent aphthous ulcerations that required discontinuation of the treatment, while the other patient’s chylothorax progressed under treatment and required surgery. In the first patient, the chylothorax remains asymptomatic and stable, so that the sirolimus treatment was not resumed.

Due to its antiangiogenic effect, treatment with IFNa-2b is also an effective treatment option in GSD. As with sirolimus, IFNa-2b is frequently used as combination therapy with bisphosphonates (Hagberg et al. 1997, Kuriyama et al. 2010, Ramaroli et al. 2019). We initiated IFNa-2b monotherapy in our very first patient. After reports of the effect of bisphosphonate treatment in GSD, we later opted for combination therapy with bisphosphonates and IFNa-2b. Due to the development of a severe hyperthyreosis, the IFNa-2b treatment had to be discontinued. As stable disease was achieved under bisphosphonate monotherapy, the IFNa-2b treatment was not resumed.

Regarding local treatment options, several authors have reported on the effectiveness of radiotherapy in GSD (Kulenkampff et al. 1990, Dunbar et al. 1993, Rauh and Gross 1997, Heyd et al. 2011, Yerganyan et al. 2015). Considering the relatively young age of GSD patients at diagnosis as well as the late effects of radiotherapy, including particularly the risk for secondary malignancies, we preferred to avoid radiotherapy in our patients.

Surgical treatment may be required in GSD patients both for the affected bones and for possible sequelae (Patel 2005). In the long bones, most authors recommend resection followed by reconstruction or endoprosthetic replacement in weight-bearing bones (Turra et al. 1990, Chan et al. 2016, Ellati et al. 2016, Liu et al. 2018).

Spinal stabilization surgery may be required in patients with spinal involvement and actual or impending neurological deficits. 2 of our patients required stabilization procedures, with one of them undergoing 2 further surgeries due to disease progression with development of cerebrospinal fluid leaks and material fatigue. Surgery might also be required in patients who develop a chylothorax—a potentially life-threatening sequela of GSD with a reported incidence of about 17% and a mortality of up to 34% (Patrick 1976, Tie et al. 1994, Ludwig et al. 2016).

A novel finding of our study concerns the QoL of affected patients. To our knowledge, no study has previously evaluated this aspect of GSD. Good to excellent MSTS, TESS, and RNL Index scores were achieved in all patients undergoing bisphosphonate treatment. Indeed, the only patient with very poor results in our cohort was the one with a contraindication to medical treatment due to a severe nephropathy, providing further evidence to the observation that systematic treatment leads to a rapid relief of symptoms (Hagberg et al. 1997).

Another somewhat surprising finding was that the patient-reported TESS and RNL Index were similar in patients with mono- and polyostotic disease, as well as in patients with and without sequelae (Table 3). We also found that younger patients tended to have higher QoL scores compared with older patients. This fact has also been observed by other authors and could be attributed to better adaptation to the disease by younger patients who grow up with it and cope better with the subsequent impairments and disabilities (Tunn et al. 2008, Heaver et al. 2016).

The low number of patients in our cohort is, naturally, a limitation of our study. However, among the approximately 300 cases reported, up to now, in the literature, our cohort represents one of the largest series treated at a single institution, reflecting the low incidence of GSD and the challenges of accruing large patient numbers.

In conclusion, due to its rare occurrence, diagnosis and treatment of GSD remain challenging. Off-label treatment with bisphosphonates appears to lead to a stable disease state in the majority of patients.

### Ethics, funding, and potential conflicts of interest

The study was approved by our local ethics committee Ethik-Kommission, Ärztekammer Westfalen-Lippe. Reference number: 2018–617–f–S. The authors received no specific funding for this work. The authors declare that they have no competing interests.

KS, DA, MW, and MM designed the study and collected the data. KS, SK, DS, JR, TV, MW, and DA were responsible for data management, data analysis, and preparation of figures. KS and DA wrote the manuscript. KS, GG, and DA helped with data analysis and with editing of the manuscript.

The authors acknowledge support from the Open Access Publication Fund of the University of Münster/Germany.

*Acta* thanks Jendrik Hardes and Ulrich Exner for help with peer review of this study.
